# Cognitive Function and Serum Hormone Levels Are Associated with Gray Matter Volume Decline in Female Patients with Prolactinomas

**DOI:** 10.3389/fneur.2017.00742

**Published:** 2018-01-29

**Authors:** Shun Yao, Jian Song, Junfeng Gao, Pan Lin, Ming Yang, Kashif Rafiq Zahid, Yan Yan, Chenglong Cao, Pan Ma, Hui Zhang, Zhouyue Li, Cheng Huang, Huichao Ding, Guozheng Xu

**Affiliations:** ^1^Department of Neurosurgery, Wuhan General Hospital, Southern Medical University, Guangzhou, China; ^2^Department of Neurosurgery, Wuhan General Hospital of PLA, Wuhan, China; ^3^Key Laboratory of Cognitive Science, College of Biomedical Engineering, South-Central of University for Nationalities, Wuhan, China; ^4^Hubei Key Laboratory of Genetic Regulation and Integrative Biology, College of Life Science, Central China Normal University, Wuhan, China; ^5^Department of Neurosurgery, Nanfang Hospital, Southern Medical University, Guangzhou, China; ^6^State Key Laboratory of Ophthalmology, Zhongshan Ophthalmic Center, Sun Yat-sen University, Guangzhou, China

**Keywords:** prolactinomas, voxel-based morphometry, gray matter volume, cognitive impairments, dysfunctional hormones

## Abstract

**Background and objective:**

Cognitive impairments have been reported in patients with hyperprolactinemia; however, there is a lack of knowledge of brain structure alterations relevant to hyperprolactinemia in prolactinomas. Thus, we aimed to identify changes in brain structure in prolactinomas and to determine whether these changes are related to cognitive performance and clinical characteristics.

**Methods:**

Participants were 32 female patients with prolactinomas and 26 healthy controls (HC) matched for age, sex, education, and handedness. All participants underwent magnetic resonance imaging brain scans, neuropsychological assessments, and clinical evaluations. Voxel-based morphometry analysis was used to identify changes in gray matter volume (GMV). Partial correlation analysis and multiple linear regression were performed to determine the relationship between GMV, cognition, and clinical characteristics.

**Results:**

Compared to HC, patients with prolactinomas demonstrated a decrease in GMV in the left hippocampus, left orbitofrontal cortex, right middle frontal cortex (MFC), and right inferior frontal cortex (IFC). In addition, patients performed worse than controls on tests for verbal memory and executive function, and this was significantly related to the GMV of the left hippocampus and right MFC, respectively. Moreover, in the patients, we found a negative relationship between serum prolactin levels and the GMV of the left hippocampus and right IFC, whereas a positive relationship was found between the GMV of the left hippocampus and serum levels of estradiol and luteinizing hormone.

**Conclusion:**

In patients with prolactinomas, specific brain structure abnormalities have been identified and are associated with cognitive impairments and dysfunctional hormones. This study enhances our understanding of brain structure changes that may occur with prolactinomas and provides novel and fundamental evidence for previous behavioral findings relevant to hyperprolactinemia.

## Introduction

Prolactin (PRL)-secreting pituitary adenomas (prolactinomas) are highly prevalent in females (1) and are the main pathologic cause of hyperprolactinemia (2). Prolactinomas may cause disturbance of endogenous hormone levels. Hyperprolactinemia inhibits pulsatile gonadotropin-releasing hormone secretion leading to direct inhibition of gonadal steroidogenesis in serum, including estradiol (E2), progesterone, follicle-stimulating hormone (FSH), luteinizing hormone (LH), and testosterone resulting in hypogonadism (2, 3). Clinical symptoms in women include galactorrhea, amenorrhea, anovulatory infertility, loss of bone mineral mass, and headache or visual disturbance due to tumor mass effects (2, 3).

In addition to these physical manifestations (2, 3), dysfunctional hormones lead to cognitive impairments (4, 5), yet, little is known about changes in brain structure in patients with prolactinomas. In the brain, hormone receptor expression has been identified in several regions and is generally located in the cell membrane or intracellularly in the nucleus, as well as in the glia, spines, and presynaptic terminals. These receptors include the PRL receptor (6), androgen receptor (7, 8), and estrogen receptor (9, 10). PRL may profoundly impact brain structure and function (6) and inhibit sex steroid hormones such as estrogen and testosterone, which also regulate neuronal morphology and numbers by influencing axonal guidance and synaptogenesis (11). Similar to prolactinomas, pregnancy often involves rapid fluctuations in hormone levels compared to non-pregnancy (12). Hoekzema et al. explored how pregnancy affects the brain and found a substantial reduction in gray matter volume (GMV) in brain areas correlated with social cognition, such as the bilateral inferior and middle frontal cortex (MFC), cingulate cortex, and hippocampus (13). However, they did not investigate the relationship between endogenous hormones and brain structure at the pre-pregnancy stage. Brain structure alterations relevant to rapid fluctuations in hormone levels have also been reported to occur during puberty (14), adolescence (15), and in pituitary adenomas with Cushing’s disease (16). However, to our knowledge, no studies have investigated the potential alterations in brain structure that may occur with prolactinomas. This is another condition in humans that involves an abnormal increase in endogenous hormone levels, which provides an intriguing opportunity to explore the effect of hyperprolactinemia on brain structure.

We hypothesized that female patients with prolactinomas would show a decline in cognitive function and structural brain alterations. We also hypothesized that there may be a relationship between cognitive performance, hormonal biochemical estimations, and structural brain changes. Thus, we performed the first cross-sectional study of patients with pituitary adenomas to explore brain structure changes using a voxel-based morphometry (VBM) approach, an unbiased whole-brain approach for the detection of structural differences in GMV. Furthermore, we determined the relationship between structural alterations, cognitive performance, and clinical characteristics.

## Materials and Methods

### Participants

**Figure 1:**
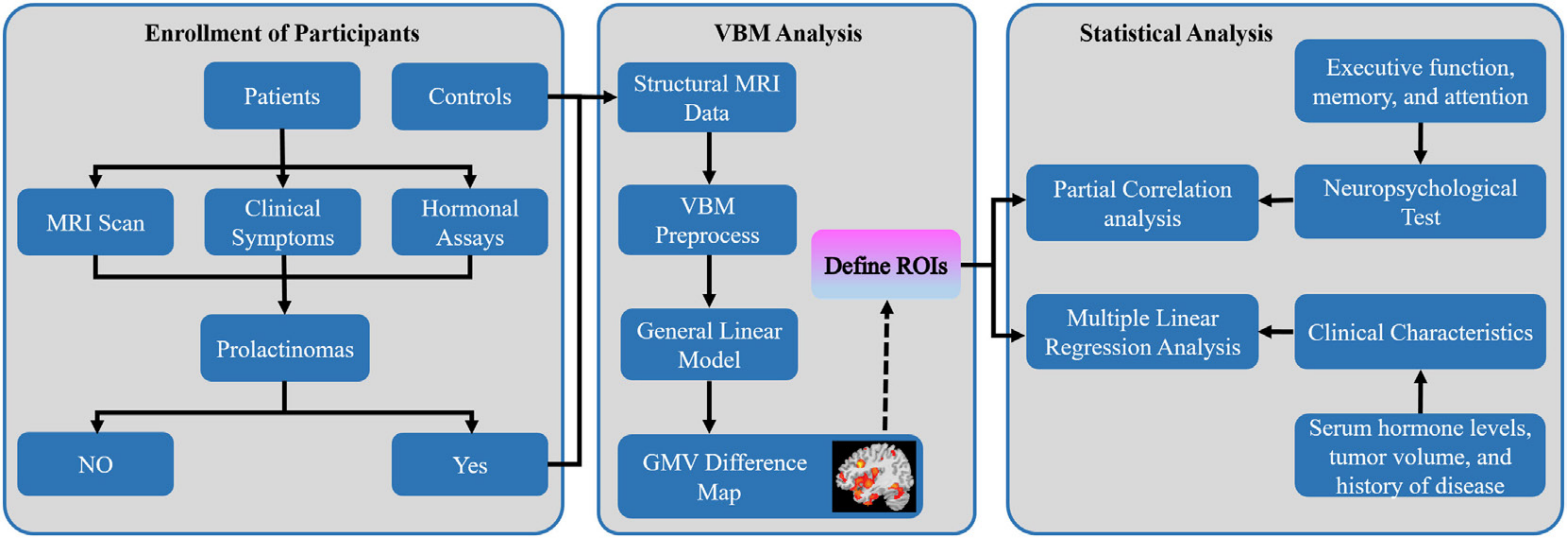
Overall workflow of the study design and pipeline of data analysis. Abbreviations: MRI, magnetic resonance imaging; VBM, voxel-based morphometry; GMV, gray matter volume; ROIs, regions of interest.

All procedures were in accordance with the Declaration of Helsinki and approved by the Ethical Committee of Wuhan General Hospital of PLA. The study protocol was fully explained and written informed consent was obtained from all participants. The inclusion criteria for patients were as follows: (1) female patients with a diagnosis of prolactinomas with hyperprolactinemia (2, 3) from the neurosurgery department of Wuhan General Hospital of PLA during April 2015 to June 2017; (2) patients who underwent magnetic resonance imaging (MRI) brain scan, neuropsychological assessments, and clinical evaluations; (3) at least 9 years education. The exclusion criteria for patients were as follows: (1) left-handed female patients; (2) patients were in pubertal stage; (3) significant visual field defect (unable to take the cognitive assessment); (4) a history of neurological or psychiatric disorders, acquired brain injury, drug or alcohol abuse [subjects who drink alcohol over 2.0 standard drinks (10 g of pure alcohol) on days and meet any 2 of the 11 criteria under the DSM-V in the past year] (17), serious smoke (subjects who have smoked over 10 cigarettes on days and manifested at least 2 of the 11 symptoms outlined in the DSM-V criteria within a 12-month period) (17), medication intake (including dopamine agonist and oral contraceptives); (5) contraindication for undergoing the MRI scan. According to above criteria, a total of 32 patients and 26 healthy controls (HC) matched for age, education, and handedness were included in this study (Figure 1).

### Neuropsychological Tests

Neuropsychological tests were administered within 2 days of the MRI scan and were carried out during a quiet period while participants were at rest. Cognitive performance was assessed by a professional psychologist. Executive function was measured using the Wisconsin Card Sorting Test (WCST)—online through a web-based program.^1^ Nonverbal memory and verbal memory were assessed using the Picture Recall Test, Visual Recognition Test, and Story Recall Test of the Wechsler Memory Scale–Revised, Chinese version (18). Attention was assessed using the Digit Span Forwards and Backwards Tests of the Wechsler Adult Intelligence Scale–Revised, Chinese version (19). Cognitive assessment took a total of approximately 30 min.

### Hormonal Assays

On the day of the MRI scan, fasting peripheral blood samples were collected by venipuncture between 8:00 a.m. and 9:30 a.m. to control for circadian variation in hormone levels. After collection, clotted and heparinized blood was delivered on ice to the clinical laboratory of Wuhan General Hospital. The “total” circulating hormone levels was obtained from serum. Serum levels of PRL (ng/ml), E2 (pg/ml), FSH (mIU/ml), LH (mIU/ml), progesterone (ng/ml), testosterone (ng/ml), growth hormone (GH) (ng/ml), thyroid-stimulating hormone (TSH, uIU/ml), and cortisol (nmol/l) were determined by chemiluminescent immunoassays (Roche, cobas^®^ 8000, Switzerland). Serum dilution for the PRL measurement (1:100) was performed to rule out the “hook effect,” if necessary.

### MRI Data Acquisition

All the participants were scanned using a 1.5T GE scanner (GE EXCITE, Milwaukee, WI, USA) with a head coil. Participants were placed in a supine position with their head fixed by cushions to minimize head motion, and all participants were provided earplugs (29 dB rating) to attenuate scanner noise. Next, high-resolution structural brain images were collected using a three-dimensional T1-weighted MRI sequences with an axial Fast Spoiled Gradient Echo sequence [repetition time (TR) = 11.5 ms, echo time (TE) = 5.1 ms, flip angle = 15°, matrix size = 256 × 256 pixels, field of view = 240 mm × 240 mm, slice thickness = 0.6 mm, and 232 contiguous transverse slices].

A neurosurgeon blinded to patients’ clinical features manually delineated the tumor volume *via* the MRIcro toolbox.^2^ None of the HC presented brain structural abnormalities as assessed by another experienced neurosurgeon, blindly.

### VBM Preprocessing

All high-resolution structural brain images were processed using a VBM analysis with the CAT12 toolbox^3^ and the SPM12 software package (Welcome Department of Cognitive Neurology, London, UK^4^) running on Matlab R2016a (Mathworks Inc., Natick, MA, USA). First, using the module “Segment Data” of CAT12, every T1-weighted image was normalized to a template space and segmented into gray matter (GM), white matter, and cerebrospinal fluid. The modulated warped GM images were then normalized to MNI-152 standard space with an isotropic voxel resolution of 1.5 mm × 1.5 mm × 1.5 mm. The images of all participants were then averaged to generate a study population-specific template. Next, using “Display one slice for all images,” we checked the data quality to figure out if some reasonable results were obtained by the segmentation and normalization procedures (if the native volume had artifacts or a wrong orientation). Using a boxplot and correlation matrices, we also checked sample homogeneity to identify outliers by visualizing the correlation between the volumes. The GM map of each individual was smoothed with an 8 mm full width at the half-maximum Gaussian kernel. Finally, using the “Estimate TIV” module, the total intracranial volume (TIV) for all the subjects was estimated. Both the automated image processing and visual check were done by two investigators blindly.

### GMV Analysis

To control the study volume for analysis, we created a study population-specific explicit optimal threshold GM mask in which only statistical analysis was performed. Using a general linear model in SPM12, we conducted an exploratory whole-brain VBM analysis to compare voxel-wise GMV difference in the study population-specific GM mask between the prolactinomas and HC with TIV, age, and education as covariates of no interest. A voxel-level false discovery rate (FDR) method with significant threshold of *p* < 0.05 was used for multiple comparisons. All the brain maps were visualized with the BrainNetViewer toolbox.^5^ Furthermore, the brain regions with significant differences in GMV were selected as regions of interest, and mean GMV values were extracted from the patients’ group to determine whether brain structural changes were correlated to cognitive performance and clinical characteristics (Figure 1).

### Statistical Analysis

Baseline clinical characteristics were presented using mean values and ranges (minimum and maximum values) for continuous variables, while the median and interquartile range for continuous variables with highly skewed distributions. The difference in continuous variables was tested using two-sample student’s *t*-test or the Mann–Whitney *U*-test due to the distributions of our data. A partial correlation analysis was used to assess correlations between the mean GMV and neuropsychological tests while adding the TIV, age, and education as additional covariates to control the confounding effects. Multiple linear regression analysis was performed to explore the independent relationship between clinical characteristics and the mean GMV with adjustment for the TIV, age, and education. Correlation analyses between GMV and neuropsychological tests, and GMV and clinical characteristics were performed as *post hoc* analyses. In addition, the Box–Cox transformation was applied for variables that did not conform to the assumptions of normality (20). Results were expressed as the beta coefficient of the linear regression model. *p* < 0.05 was considered statistically significant. All statistical analyses were performed with EmpowerStats^6^ and R (R Foundation for Statistical Computing, Vienna, Austria).

## Results

### Demographic, Clinical, and Neuropsychological Data

Demographic and clinical data for all participants is presented in Table 1. No significant differences were observed between patients with prolactinomas and matched HC in age (*p* = 0.424) and education (*p* = 0.270). However, we did find a significant difference in the TIV (*p* = 0.001). Neuropsychological results are shown in Table 2. Compared to HC, patients with prolactinomas performed significantly worse on the tests of verbal memory (Story Recall, *p* = 0.023) and executive function (WCST, *p* = 0.008).

**Table 1:** Demographic and clinical characteristics: prolactinomas patients and healthy controls (HC).

	Prolactinomas (*n* = 32)	HC (*n* = 26)	*p-*Value
Age (years)	46.22 (28.00–54.00)	45.88 (33.00–53.00)	0.424^a^
Education (years)	11.41 (9.00–17.00)	11.92 (9.00–11.00)	0.270^a^
History of onset (months)	6.00 (2.25–42.00)	NA	
Tumor volume (cm^3^)	3.15 (1.50–7.95)	NA	
TIV (cm^3^)	1,416.69 (1,200.59–1,738.50)	1,525.21 (1,310.66–1,794.57)	0.001^b^
Serum PRL (ng/ml)	98.86 (64.47–242.83)	NA	
Serum E2 (pg/ml)	42.78 (7.04–92.25)	NA	
Serum progesterone (ng/ml)	0.24 (0.08–0.94)	NA	
Serum FSH (mIU/ml)	7.46 (4.58–18.33)	NA	
Serum LH (mIU/ml)	4.75 (1.04–20.11)	NA	
Serum testosterone (ng/ml)	0.14 (0.06–0.30)	NA	
Serum GH (ng/ml)	0.65 (0.16–2.14)	NA	
Serum TSH (mIU/ml)	1.84 (1.09–2.74)	NA	
Serum cortisol (nmol/l)	390.70 (259.13–460.78)	NA	

*^a^Mann–Whitney U-test*.

*^b^Student’s *t*-test (two-tailed)*.

*TIV, total intracerebral volume; PRL, prolactin; E2, estradiol; FSH, follicle-stimulating hormone; LH, luteinizing hormone; GH, growth hormone; TSH, thyroid-stimulating hormone*.

**Table 2:** Group difference in neuropsychological tests.

	Prolactinoma patients (*n* = 32)	Healthy controls (*n* = 26)	*p-*Value
**Executive function**			
WCST, median (IQR)	4.50 (4.00–5.00)	5.00 (5.00–6.00)	0.008^a^
**Non-verbal memory**			
Picture recall	8.00 (7.00–10.75)	10.00 (8.00–11.25)	0.178^a^
Visual recognition	9.00 (7.00–11.00)	9.00 (8.75–11.00)	0.106^a^
**Verbal memory**			
Story recall	7.75 (2.00–16.00)	9.73 (5.00–17.00)	0.023^b^
**Attention**			
Digit span forward	8.00 (6.00–10.75)	9.00 (8.00–11.25)	0.088^a^
Digit span backward	8.00 (5.00–10.00)	7.50 (6.75–10.00)	0.305^a^

*^a^Mann–Whitney U-test*.

*^b^Student’s *t*-test (two-tailed)*.

*Values of each neuropsychological test reflect the degree of cognitive performance. The higher values, the better performance in executive function, non-verbal/verbal memory, and attention; while the lower values, the worse performance in cognitive function*.

*WCST, Wisconsin Card Sorting Test; IQR, interquartile range*.

### GMV Differences

**Figure 2:**
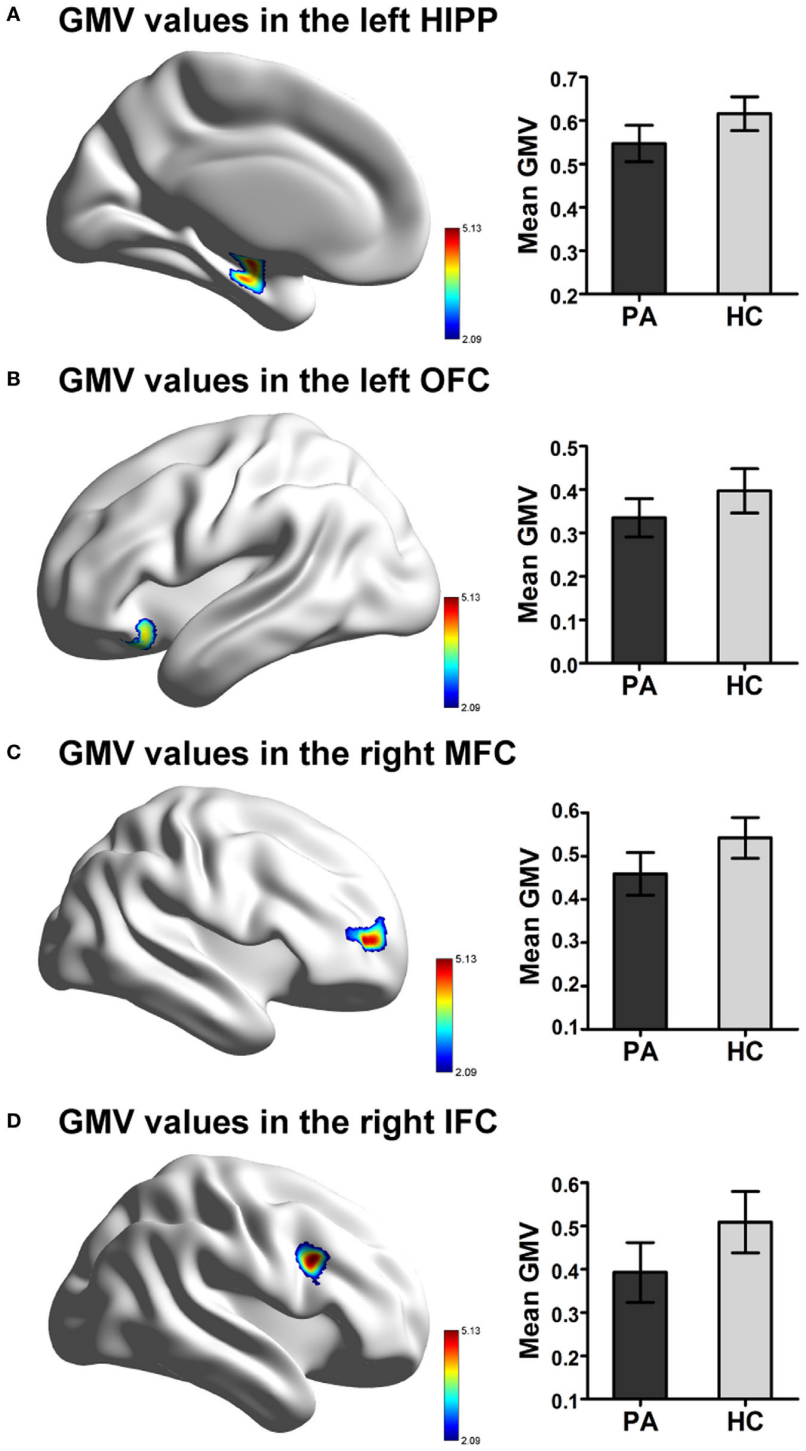
Significant difference in GMV in patients with prolactinomas (PA) compared to healthy controls (HC). **(A)** Decreased GMV were presented in the left HIPP. **(B)** Decreased GMV were presented in the OFC. **(C)** Decreased GMV were presented in the right MFC. **(D)** Decreased GMV were presented in the IFC (FDR corrected, *p* < 0.05). Abbreviations: GMV, gray matter volume; HIPP, hippocampus; OFC, orbitofrontal cortex; MFC, middle frontal cortex; IFC, inferior frontal cortex; FDR, false discovery rate.

A voxel-level FDR correction for multiple comparisons (*p* < 0.05) in the exploratory whole-brain analysis confirmed significant volumetric differences in patients with prolactinomas, in regions such as the left orbitofrontal cortex (OFC), the left hippocampus, the right MFC, and the inferior frontal cortex (IFC) (Table 3; Figure 2).

**Table 3:** GMV differences between patients with prolactinomas and healthy controls (*p* < 0.05, FDR corrected).

Brain regions	Hem	BA	MNI coordinate of peak			Cluster size (voxels)	*T*-value
			*x*	*y*	*z*		
HIPP	L		−27	−13.5	−18	81	5.04
OFC	L	47	−40.5	18	−15	72	4.65
MFC	R	10	30	55.5	7.5	57	5.13
IFC	R	44	40.5	13.5	30	128	5.13

*GMV, gray matter volume; HIPP, hippocampus; OFC, orbitofrontal cortex; MFC, middle frontal cortex; IFC, inferior frontal cortex; Hem, hemisphere; BA, Brodmann area; MNI, Montreal Neurological Institute; FDR, false discovery rate; L, left; R, right*.

### GMV, Cognitive Performance, and Clinical Characteristics

**Figure 3:**
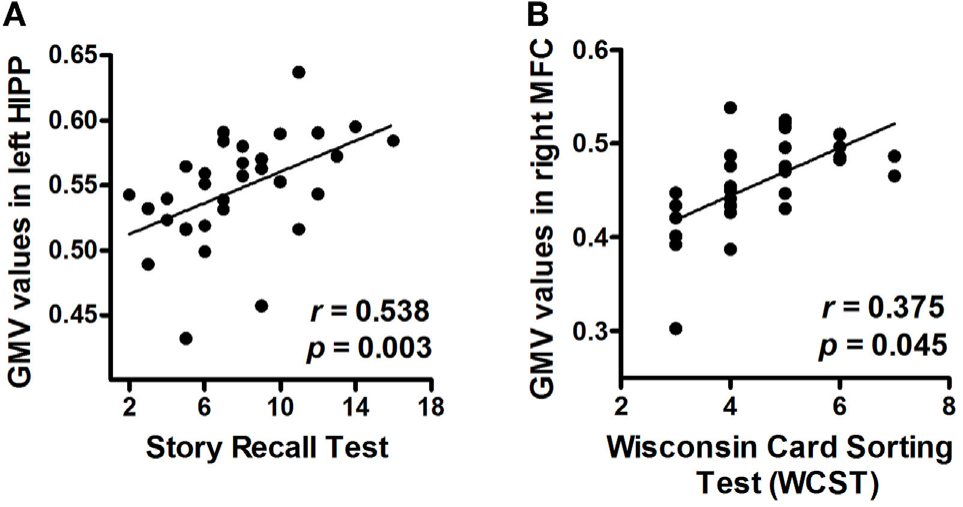
Correlations between neuropsychological measurements and mean GMV values (*post hoc* analyses). **(A)** Correlation between the Story Recall Test performance and mean GMV values in the left HIPP. **(B)** Correlation between the Wisconsin Card Sorting Test (WCST) performance and mean GMV values in the right MFC. Abbreviations: GMV, gray matter volume; HIPP, hippocampus; MFC, middle frontal cortex.

We found that patients with greater volumes of the left hippocampus and right MFC performed better on tests for verbal memory (Story Recall Test: *r* = 0.538, *P*_FDR-corrected_ = 0.003) and executive function (WCST: *r* = 0.375, *P*_FDR-corrected_ = 0.045), respectively (Figures 3A,B).

Multiple linear regression analysis of serum levels of PRL, E2, FSH, LH, progesterone, testosterone, GH, TSH, and cortisol, tumor volume, and history of disease onset as dependent variables, and the mean GMV of left hippocampus, left OFC, right MFC, and right IFC as independent variables in the prolactinomas group, showed a negative relationship between serum PRL levels and mean GMV of the left hippocampus (*p* < 0.01) and right IFC (*p* < 0.05), whereas there was a positive relationship between the mean GMV of the left hippocampus and serum levels of E2 (*p* < 0.01) and LH (*p* < 0.05). No significant relationship was found between the mean GMV and any of the other clinical measurements (Table 4).

**Table 4:** Multiple linear regression analyses of GMV and clinical characteristics in patients with prolactinomas.

Clinical characteristics	The mean GMV of brain alterations			
	Left HIPP	Left OFC	Right MFC	Right IFC
PRL	−0.022^b^	0.001	−0.007	−0.037^a^
E2	0.013^b^	0.000	0.003	0.006
Progesterone	0.004	−0.002	−0.001	0.001
FSH	0.006	−0.011	−0.011	0.015
LH	0.009^a^	−0.003	0.002	0.013
Testosterone	0.007	−0.003	−0.006	−0.001
GH	0.004	0.004	−0.002	0.005
TSH	−0.004	−0.017	−0.006	0.006
Cortisol	0.000	0.000	0.000	0.000
Tumor volume	−0.004	0.009	−0.006	0.005
History of disease onset	0.000	0.003	0.000	0.001

*^a^p < 0.05, adjusted for total intracranial volume, age, and education*.

*^b^p < 0.01, adjusted for total intracranial volume, age, and education*.

*Variables underwent Box–Cox transformation: PRL, E2, progesterone, FSH, LH, testosterone, GH, TSH, tumor volume, and history of disease onset*.

*The multiple linear regression model was adjusted for total intracranial volume, age and education*.

*The correlation analyses conducted between GMV and clinical characteristics were performed as post hoc analyses*.

*GMV, gray matter volume; HIPP, hippocampus; OFC, orbitofrontal cortex; MFC, middle frontal cortex; IFC, inferior frontal cortex; PRL, prolactin; E2, estradiol; FSH, follicle-stimulating hormone; LH, luteinizing hormone; GH, growth hormone; TSH, thyroid-stimulating hormone*.

## Discussion

In this study, we found that patients with prolactinomas showed GMV decline in the left hippocampus and prefrontal cortex including the left OFC, the right MFC, and the right IFC, suggesting brain structure damages in patients with prolactinomas. The decline of GMV in left hippocampus and right MFC were significantly correlated with cognition deficits in verbal memory and executive function, respectively. In addition, serum hormone levels including PRL, E2, and LH, presented independent relationships with the mean GMV. These findings provide fundamental evidence for previous reports of cognitive dysfunction in a population with high PRL serum levels (4, 21, 22).

Changes in GM extracted from high-solution MRI images can reflect changes in the number of synapses, glial cells, neuronal cell bodies, dendrites, myelinated and unmyelinated axons, and capillaries. Thus, the reductions in GMV shown in our study may at least be related to decreasing in partial components. Brain structure is the foundation of brain function (23). In this study, GMV changes were found in the left hippocampus and prefrontal cortex, regions generally involved high-level cognitive functions. Memory impairments are frequently reported with hyperprolactinemia including decline in short-term memory, verbal or non-verbal memory (4, 21). Consistently, we found that patients with prolactinomas showed a sharply decreased GMV in left hippocampus that was correlated to verbal memory deficits. The hippocampus is an important brain structure for information consolidation from short-term memory to long-term memory and non-verbal memory in both animals and humans (24, 25). Scientists have also suggested that the hippocampus is part of the medial temporal lobe memory system responsible for declarative memory, which is often related to any kind of memory that can be explicitly verbalized in the recall paradigms (24, 25), such as story recall paradigm in our study. In recall response generation, specifically recollecting verbal or non-verbal information, left-lateralization is typically related to language processing (26). Previous studies have shown that hyperprolactinemia that occurs frequently in the population of schizophrenia also affects the structure of the hippocampus (27). Moreover, the left hippocampus seems to be more vulnerable to high PRL levels than that of the right, which is consistent with our results (28).

Impairments of executive function have also been reported to occur with hyperprolactinemia (4, 21). These cognitive dysfunctions are involved mainly in the prefrontal cortex (29, 30), a region that we also found abnormalities in patients with prolactinomas. As a sub-region of the prefrontal cortex, the anterior part of right MFC (BA 10) is an area of the dorsolateral prefrontal cortex (DLPFC) and has connections with the OFC, thalamus, hippocampus, caudate nucleus, and primary and secondary association regions of the neocortex. The DLPFC plays an important role in executive function, such as planning, working memory, and cognitive flexibility (31), which are measured by the WCST with adequate sensitivity (32). Interestingly, our study also showed a close relationship between the mean GMV of the right MFC and executive function in patients with prolactinomas. The pars opercularis of the right IFC, also known as Brodmann’s Area 44 (corresponding to left Brodmann’s Area 44, commonly known as Broca’s area), is characterized as a pivotal region for inhibitory control, implemented by the front-basal-ganglia circuits (33). However, we did not find a significant relationship between the GMV of right IFC and the WCST performance, which may be due to its limited specificity to the localization of subareas of the prefrontal cortex (34). Besides this, executive function involves widespread brain areas including the prefrontal cortex, caudate nucleus, and subthalamic nucleus (35, 36). However, substantial evidence has shown that during Go/No-Go tasks, the right IFC is the region most frequently activated (33). Importantly, our team has performed Go/No-Go tasks for the patients with pituitary adenoma and demonstrated that the amplitude of N2d and P3d over the frontal electrode sites was more weaker and delayed compared to HC. These results indicated a decline at earlier and later stages of inhibitory processes in these participants and implicate malfunction of inhibitory controls (37). In addition, lesion or interference studies of the OFC have shown its widespread functions in response to inhibition, flexible associative encoding, emotion, and reward. However, these existing interpretations are not freestanding or the core function of the OFC (38). This may explain why we did not find a significant relationship between the OFC and cognitive performance in our study.

Previous studies have also established that changes in brain structure and impairments in cognition occur in patients with prolactinomas. We further investigated the underlying relationship between abnormalities in brain structure and clinical characteristics, while taking into consideration that the hippocampus and prefrontal cortex are target brain structures of hormonal action (6, 8, 9, 39). Further multiple linear regression analysis revealed that the left hippocampus and right IFC were adversely influenced by high serum PRL levels, whereas the left hippocampus was positively associated with serum levels of E2 and LH. Previous studies in rodents have shown that proper levels of PRL plays an important role in preventing a stress-induced decrease of adult hippocampal neurogenesis and exerts neuroprotection against excitotoxicity in hippocampal neurons mainly *via* PRL receptors (40, 41). Nevertheless, if the concentration of PRL is abnormally high, it may adversely and dramatically affect cognitive processing (4, 21, 42, 43). Consistent with our results, previous studies have found that E2 may be synthesized in the hippocampus and perform neuroprotective effects that are beneficial to memory by regulating spines and synapse in the hippocampus (9, 39). Previous studies have also shown that the LH receptor is present in the hippocampal formation and regulates age-related cognitive decline (44, 45). However, we found a positive relationship between LH serum levels and the structure of the hippocampus, which may be attributed to the underlying inhibitory effect of the high PRL levels in serum (46). Moreover, we also found that serum PRL levels were correlated to the right IFC, suggesting that higher PRL levels may have a detrimental effect on executive function. This effect may be indirectly mediated by the dopamine (47), or directly regulated by the high concentration of PRL in the right IFC (6). Another interesting finding was that the TIV in the patients with prolactinomas was significantly lower than HC. Previous studies have found that high serum levels of PRL could lead to cognitive detriments such as memory and executive function, which are closely linked to brain structures (4, 13, 21). In addition, hyperprolactinemia can inhibit sex steroid hormone release, which is well known to play a crucial role in regulating GM architecture and brain size in humans (48, 49). Thus, this finding provides evidence that patients with prolactinomas suffer from not only the GM loss in regional areas but also TIV. However, the underlying mechanism of structural alterations relevant to dysfunctional hormones is not yet clearly understood due to a limited number of *in vivo* or *ex vivo* studies in animals.

Although we report several novel findings, there are some limitations of our study. First, scans were done on a 1.5T scanner, which has a lower resolution as compared to newer 3T or later models. Nevertheless, all the quality controls for structural MR images were processed using the highest standards using the “Display one slice for all the images” module and the “Check sample homogeneity” module in the CAT12 toolbox. Second, although we have enrolled our participants with very strict criteria, the small sample size may have influenced our results. Finally, the cross-sectional design used, does not allow us to determine causality between hyperprolactinemia, changes in brain structure and cognitive dysfunctions. Regardless, our results provide novel insights into the profound impact of hyperprolactinemia on the cerebral GM structure in patients with prolactinomas.

## Conclusion

In conclusion, this study demonstrates that female patients with prolactinomas suffer cognitive impairments and GM loss in the left hippocampus and prefrontal cortex. In addition, there are important links between hormone levels, cognitive performance, and GMV. Our findings suggest that dysfunctional hormones in serum may have a pivotal role in GM decline and cognitive impairments. Further studies with a larger sample size and longitudinal observations are needed to investigate the relationship between dysfunctional hormone levels, brain structure and cognitive performance over time in patients with prolactinomas.

## Ethics Statement

All procedures were in accordance with the Declaration of Helsinki and approved by the Ethical Committee of Wuhan General Hospital of PLA. The number of the approved ethical statement is “[2014] 024-1.” All subjects were fully informed of the nature of the study protocol and all gave their written consents regarding participation.

## Author Contributions

GX, JS, PL, and JG were responsible for the study design, major research funding, interpreting the data, and revising the first draft of the manuscript. SY performed the brain mapping, VBM analysis, statistical analysis, and drafted the manuscript. MY and HZ attributed to MRI data acquisition, quality control assessment of all the MRI data, manually delineate the tumor volume, check brain structural abnormality and the study design. YY, CC, PM, CH, and HD took part in recruiting subjects, acquiring MRI data, collecting clinical data, and performing neuropsychological assessments (CC, PM). KZ and ZL assisted SY to carry out the brain mapping, processing of VBM analysis, write the section of VBM analysis, and revise the drafts of the first manuscript. All authors critically reviewed and approved the final manuscript.

## Conflict of Interest Statement

All the authors declare that the research was conducted in the absence of any commercial or financial relationships that could be construed as a potential conflict of interest.

## Footnotes

^1^http://www.dweipsy.com/lattice/.

^2^http://www.mccauslandcenter.sc.edu/crnl/tools/.

^3^http://dbm.neuro.uni-jena.de/cat/.

^4^http://www.fil.ion.ucl.ac.uk/spm/software/spm12/.

^5^http://www.nitrc.org/projects/bnv/.

^6^www.empowerstats.com.
